# Beyond sleep duration: Sleep timing as a risk factor for childhood obesity

**DOI:** 10.1111/ijpo.12698

**Published:** 2020-07-29

**Authors:** Hanna F. Skjåkødegård, Yngvild S. Danielsen, Bente Frisk, Sigurd W. Hystad, Mathieu Roelants, Ståle Pallesen, Rachel P. K. Conlon, Denise E. Wilfley, Petur B. Juliusson

**Affiliations:** 1 Department of Clinical Science, University of Bergen, Bergen, Norway; 2 Department of Clinical Psychology, University of Bergen, Bergen, Norway; 3 Department of Health and Functioning, Western Norway University of Applied Sciences, Bergen, Norway; 4 Department of Physiotherapy, Haukeland University Hospital, Bergen, Norway; 5 Department of Psychosocial Science, University of Bergen, Norway; 6 Department of Public Health and Primary Care, KU Leuven, University of Leuven, Leuven, Belgium; 7 Norwegian Competence Center for Sleep Disorders, Haukeland University Hospital, Bergen, Norway; 8 Department of Psychiatry, University of Pittsburgh School of Medicine, Pittsburgh, Pennsylvania; 9 Department of Psychiatry, Washington University School of Medicine, St. Louis, Missouri; 10 Department of Health Registries, Norwegian Institute of Public Health, Bergen, Norway; 11 Department of Pediatrics, Haukeland University Hospital, Bergen, Norway

**Keywords:** childhood obesity, sleep, sleep timing, social jetlag

## Abstract

**Background:**

Ample evidence attests to the relationship between short sleep duration, sleep problems and childhood obesity. However, few studies have examined the association between sleep timing and obesity in children.

**Objectives:**

To investigate how sleep duration, problems and timing relate to obesity and obesogenic behaviours in children.

**Methods:**

Eighty-five children (58.8% girls) with severe obesity and mean (SD) age of 12.1 (2.9) years, were matched by age and sex with peers with normal weight (n = 85,12.0 [2.8] years). Sleep and moderate-to-vigorous physical activity (MVPA) were measured via accelerometer for seven consecutive days. Children self-reported emotional eating on the Dutch eating behavior questionnaire. Parents reported children’s screen time and sleep problems.

**Results:**

Children with severe obesity had significantly later mean mid-sleep time, overall (36 minutes later, *P* < .001), on school nights (36 minutes later, *P* < .001) and weekend nights (39 minutes later, *P* = .002) compared to children with normal weight. Children with obesity had more sleep problems (*P* = .030), but no differences emerged in sleep duration or social jetlag. After adjusting for demographic factors, mid-sleep time was positively related to screen time (*P* = .030). Mid-sleep time and sleep duration were inversely related to time in MVPA (*P*s ≤ .041). There were no other significant associations between the sleep variables and the obesogenic behaviours.

**Conclusions:**

Later sleep timing was related to obesogenic behaviours in children and may represent an obesity risk factor.

## 1 | INTRODUCTION

Childhood obesity is a complex, multicausal health issue with major consequences for both the individual and society.^1^ The detection of risk factors associated with weight gain is fundamental to offer adequate prevention and treatment. Short sleep, as well as sleep problems, have for some time been recognised as risk factors for obesity.^2^ The majority of research on sleep and childhood obesity has focused on sleep duration.^3–5^ Accordingly, an increasing number of studies have reported cross-sectional and longitudinal associations between short sleep duration and childhood obesity, suggesting sleep duration to be an independent and modifiable risk factor for the condition.^3–5^ However, recent studies indicate that, in addition to sleep duration, other aspects of sleep needs to be taken into consideration to provide a more comprehensive understanding of how sleep contributes to the development and maintenance of obesity in children.^2,6,7^

Late sleep timing (i.e., when sleep occurs) and social jetlag (usually defined as the difference in mid-sleep time between weekdays and weekends^8^) have recently been suggested as unique contributors to obesity risk in school-aged children and adolescents, independent of sleep duration.^9–13^ Late sleep timing has specifically been associated with increased weight, unhealthy eating habits, decreased physical activity levels and more screen time.^9–11,13–17^ A small cross-sectional study in adolescents with obesity (n = 26) found that later sleep timing was associated with a higher caloric intake and more screen time independent of total sleep duration.^15^ A recent study on treatment seeking adolescents with overweight and obesity found that later weekend bedtimes and greater social jetlag were significantly associated with severity of overweight.^6^ Further, social jetlag has been associated with metabolic changes and obesogenic behaviours such as more screen time, less physical activity and emotional eating.^6,10,12,18–21^

Both biological and behavioural causes of weight gain seem to be associated with delayed and shifted sleep timing, but there is dearth of knowledge regarding our understanding of these associations.^16,22,23^ It is possible that mistiming of sleep promotes circadian misalignment and, eventually, increases risk of developing obesity.^23,24^ Therefore, a focus on alignment of sleep timing with underlying circadian rhythms could enhance paediatric obesity prevention and treatment.^23^ Only a handful of previous studies have so far used objective sleep measures to investigate the sleep-obesity relationship.^14,17,20,25^ Subjective sleep measures are associated with various biases,^26–28^ therefore, more studies using objective sleep measures in a natural home environment, such as accelerometers, are needed. The present cross-sectional study adds to current research by using accelerometers (instead of self- or parent-reported measures) to assess sleep timing and social jetlag in school-aged children and adolescents.

The aims of this study were to investigate how children’s sleep duration, sleep timing (including social jetlag) and sleep problems were linked to obesity and behavioural factors known to cause obesity in children. We hypothesised that in addition to sleep duration, delayed sleep timing and social jetlag were also independently related to behavioural factors that place school-aged children at a greater risk for developing obesity.

## 2 | METHODS

### 2.1 | Participants

In total, cross-sectional data from 170 children (median age 12.4 years, range 5.8–17.1 years) were collected between February 2014 and March 2018; 85 children (50 girls) with severe obesity and 85 children with normal weight, matched by age, sex and season of accelerometer measurement (April-September vs October-March). Participants with severe obesity were recruited from the Obesity Outpatient Clinic, Haukeland University Hospital, Bergen, Norway via referral from general practitioners. The criterion for clinic admission was a body mass index (BMI) above the International Obesity Task Force (IOTF) cut-off for severe obesity (≥ IOTF 35),^29^ or above the cut-off for obesity (≥ IOTF 30) in the presence of weight-related comorbidity (e.g., psychosocial problems or emergence of cardio-metabolic risk factors). The group with normal weight (BMI ≤ IOTF 25) was recruited from randomly selected schools in the Bergen municipality. Stratified random sampling ensured that the comparison group were matched for age and sex.

Written informed consent was obtained from the parent(s) and from participating children above 12 years of age. The study was approved by the Regional Committee for Medical and Health Research Ethics in Western Norway (number 2013/1300) and was registered at http://clinicaltrials.gov (NCT02687516).

### 2.2 | Anthropometric measures

Weight status was assessed by the BMI (kg/m^2^) calculated from measured height and weight and converted to BMI z-scores using the Norwegian growth references.^30^ For the group of children with severe obesity, height and weight were measured by trained assessors at the Obesity Outpatient Clinic. Height was measured to the nearest 0.1 cm with a wall-mounted electronic stadiometer (Seca 264, Seca, Hamburg, Germany), and weight was measured to the nearest 0.1 kg using a digital scale (InBody 720, Biospace, Seoul, Korea). For the group of children with normal weight, measurements were collected by a trained assessor during regular school hours in their school nurse’s office. Standing height was measured with a Harpenden portable stadiometer (Crosswell, UK) to the nearest 0.1 cm. Weight was measured on a calibrated Seca personal digital scale (Hamburg, Germany) to the nearest 0.1 kg. Participants in both groups were measured wearing light indoor clothing (excluding shoes and socks).

### 2.3 | Sleep measures

Sleep was assessed using the Actiwatch 2 (Philips Respironics, BEND, OR). Actiwatch 2 devices are wrist-worn accelerometers with a light sensor and an event marker and record all uni-axial movement over 0.05G.^31^ Data was collected using 30-second epochs, each scored as either “wake” or “sleep” based on a medium sensitivity threshold. Medium sensitivity threshold has shown to yield the least biassed estimates of wakefulness, total sleep time and wake after sleep onset in school-aged children.^31,32^ The Actiwatch 2 was worn on the wrist of the non-dominant arm for seven consecutive days. Participants were instructed to press the event marker when switching off the light at night and when waking up in the morning. Actiwatch 2 is validated, both in clinical sleep laboratories and in natural home environments, and is commonly used for sleep research in children aged 3 to 18 years.^32–34^

Respironics Actiware software version 6.0.9 was used to calculate sleep statistics. The rest interval (time in bed) associated with the main sleep period in the 24-hours day was manually set according to a standardises scoring protocol.^31^ To ensure inter-rater reliability, 30% of the actigraphy recordings were scored twice, by two independent observers, and compared in terms of total time in bed and total sleep time. The percentage of agreement among observers were 99.6% for total time in bed and 99.9% for total sleep time. After the rest interval was manually defined, the proprietary software automatically produced sleep statistics within the interval. The variables sleep onset time and wake up time (sleep offset) were used in our analyses.

### 2.4 | Sleep duration

When the rest interval was defined, the software automatically detected time spent asleep within the rest period. Average sleep duration for 7 days, average sleep duration on school nights (Sunday through Thursday nights) and weekend nights (Friday and Saturday nights) were used in the analyses. To be included, the participant had to have completed recordings of at least 5 days (out of 7 days) including at least three school nights and two weekend nights. We also categorised sleep duration for 7-day average, and school and weekend nights separately, based on recommendations from the National Sleep Foundation (NSF).^35^ The NSF recommends 9 to 11 hours of sleep for children aged 6 to 13 and 8 to 10 hours of sleep for adolescents aged 14 to 17, respectively, while <7 hours is “not recommended” for either age group.^35^

### 2.5 | Mid-sleep time

Sleep timing was operationalised as mid-sleep time according to the formula: (sleep onset time + sleep offset time)/2. The mid-sleep time point of each individual child was calculated as a 7 day average as well as for school nights and weekend nights separately. For participants with six or five nights of recordings, the average of these nights was used. To be included in weekend night’s analyses, two nights of recordings were needed. Further, sleep onset time and final wake up time are reported to provide additional information about sleep timing. Sleep during daytime was not assessed in the study.

### 2.6 | Social jetlag

Social jetlag quantifies the discrepancy between circadian time and social time^19^ and was operationalised as the difference between the mean mid-sleep time point on school nights and the mean mid-sleep time point on weekend nights.

### 2.7 | Physical activity measures

Physical activity level was objectively assessed using data from the same device (Actiwatch 2) during daytime (8 am-9 pm). Data were downloaded using Respironics Actiware software version 6.0.9 and transferred to Microsoft Excel 2016 for further processing with a tailored-made algorithm to divide the collected activity data into different activity levels based on previously used and validated cut-off values.^36^ The cut-off values were: light intensity (160–523 counts/30 second-epochs), moderate intensity (524–811 counts/30 seconds-epochs) and vigorous intensity (>812 counts/30 second-epochs).^36^ Physical activity level was operationalised as the percentage of time spent in moderate-to-vigorous physical activity (MVPA). Participants had to have at least 10 hours of wear time between 8 am to 9 pm and at least 4 days of recorded data to be included in the physical activity analysis.^36^ Sleep during this period was automatically coded as either non-wear time or sedentary behaviour (movement while sleeping) by the tailor-made algorithm.

### 2.8 | Emotional eating

#### 2.8.1 | The Dutch eating behavior questionnaire child version

Emotional eating was assessed with the Dutch eating behavior questionnaire child version (DEBQ-child).^37^ The DEBQ-child is a 33-item self-report questionnaire and consists of three sub-scales: emotional eating, external eating and restrained eating. All items are rated on a five-point scale ranging from never (1) to very often (5). For each subscale a mean score is calculated, with the following clinical cut-off values for emotional eating: >2.22 (boys) and >2.36 (girls). Participants were grouped according to whether they were below or above the clinical cut-off value. The DEBQ-child is increasingly used for research on children with obesity and has adequate internal consistency, test-retest reliability, factorial validity and dimensional stability for measuring disordered eating behaviours in children aged 7 to 17 years.^37,38^ In the current study, the Cronbach alpha coefficient for the emotional eating subscale was .96, suggesting a high internal consistency of the scale in the current sample. The questionnaire was completed at the Obesity Outpatient Clinic by children with obesity and at the school nurse office by children with normal weight.

#### 2.8.2 | Demographic information

Family structure, parental education levels, parental employment, child sleep problems and child daily screen time were measured with a parental questionnaire. Family structure was evaluated with the following questions: “Are both parents living together” and “Do the child live together with siblings”. Parental education level was categorised as either low (≤3 years of high school), intermediate (≤4 years of college/university) or high >4 years of college/university). Sleep problems were identified with the following question: “Has the child in any period experienced sleep problems”, with the following response categories: “never”, “before starting elementary school but not now”, “after starting elementary school but not now», and “current sleep problems”. Participants were grouped according to whether they reported current sleep problems or not. Habitual screen time was rated on a scale from 0 (no screen time) to 5 (>4 hours of screen time). The questionnaires were completed at the Obesity Outpatient Clinic by the parents of children with obesity and sent by mail to the parents of children with normal weight.

### 2.9 | Statistical analyses

Data were analysed with IBM SPSS version 25 (IBM Corp., Armonk, NY). Descriptive statistics of continuous variables are given by the mean and SD, and of categorical variables by the frequency and percentage. Demographic variables in the normal weight and obesity groups were compared with independent sample *t* tests and chi-square tests of independence. Sleep parameters were compared between groups with independent sample *t* tests. In addition to group mean differences on the measurement scale, we also calculated the effect sizes (Cohen’s *d*). An effect size of 0.2 is considered small, 0.5 medium and 0.8 large,^39^ respectively. We used hierarchical multiple regression analyses to regress screen time and MVPA on mid-sleep time, sleep duration, social jetlag and sleep problems adjusted for group (normal weight or severe obesity), age, sex, living situation (operationalised as parents living together or not) and parental education level. Group, age, sex, living situation and parental education level were entered in Step 1 of the analysis, while the focal predictors were entered in Step 2. Parental education was entered as two dummy variables with the low education group as reference category. Finally, we included interaction terms between the group variable and the four focal predictors in a final, third step. The continuous predictors (mid-sleep time, sleep duration and social jetlag) were all mean centred prior to computing the interaction terms.

A logistic regression analysis was used to examine the association between sleep duration, mid-sleep time, social jetlag and sleep problems with emotional eating, adjusted for group, age and sex.

### 2.10 | Power estimates

The required sample size was determined with G*Power, version 3.1.3.^40^ An *α* of .05 (two-tailed) and power (1−*β*) of .80 was used to determine statistical significance. For the group comparison, a minimum of 51 individuals in both groups of children (with obesity/normal weight) is required to detect a medium effect size (Cohen’s *d* = 0.50) with a significance level of .05, a power of 80%. The present sample size of 85 children per group allows to detect effect sizes of 0.4 onwards.

### 2.11 | Missing data

Because of some missing data, the number of children with useable data observations ranged from 124–170 in the different analyses.

Of the 170 participants, 168 (98.8%) provided valid accelerometer data on sleep and were included in the analyses. Of these, 154 provided valid recordings for seven consecutive days and, 14 for 6 or 5 days. Of the 168 eligible participants, two did not have sufficient actigraphy data for weekend nights, reducing the sample to 166 (97.6%) for these analyses. Further, 16 (9.4%) participants in total, 12 from the group of children with obesity and four of the normal weight peers, were excluded from the analyses involving MVPA because they had less than 10 hours of wear time between 8 am to 9 pm and/or less than 4 days of recording. All parents of children with obesity and 65 out of 85 (76.5%) of parents of children with normal weight completed the questionnaire on demographic data, sleep problems and screen time. Seventy-seven out of 85 (90.6%) participants in the group of children with obesity and all participants in the group of peers with normal weight completed the DEBQ-child questionnaires on emotional eating.

## 3 | RESULTS

### 3.1 | Demographical and clinical characteristics of the sample

The groups of children with obesity and their peers with normal weight were balanced in terms of age, sex and ethnicity (Table 1). However, children with normal weight more often lived with both parents and with siblings, and their parents were more often employed and higher educated. Overall, 92.3% of the children did not meet the NSF age-appropriate sleep recommendation, while 13.1% had an average sleep duration classified as not recommended (<7 hours). For school nights the percentage not meeting the recommendations where 91.7%, with 22.6% sleeping less than 7 hours. For weekend nights the percent not meeting the recommendations where 69.9%, with 9.0% sleeping less than 7 hours.

### 3.2 | Sleep behaviour: Comparison of children with severe obesity and normal weight

Children with severe obesity had a significantly later mid-sleep time, both overall (on average 36 minutes later, *P* < .001) as well as on school nights (36 minutes later, *P* < .001) and weekend nights (39 minutes later, *P* = .002) separately (Table 2). In addition, children with obesity had more often sleep problems (28.0% vs 20.9% in normal weight children, *P* = .03). However, sleep duration and social jetlag did not significantly differ between groups (Table 2).

### 3.3 | Association between sleep behaviour and obesogenic behaviours

In the total sample, mid-sleep time point and sleep duration significantly correlated with screen time and MVPA (*P*s ≤ .01). Social jetlag was significantly correlated with screen time (*P* = .02), but not with MVPA (*P* = .08). Sleep problems were not significantly correlated with either screen time (*P* = .07) or MVPA (*P* = .30). The results are summarised in Table 3.

In the hierarchical model, age, sex, group, living situation and parental education level entered at Step 1 combined explained approximately 21% of the total variability in screen time use (*R*^2^ = .212, *F*[8, 127] = 4.27, *P* < .001). Of our focal predictors entered in Step 2, only mid-sleep time was significantly related to screen time use (*β* = .26, *P* = .03). Combined, adding mid-sleep time, sleep duration, social jetlag and sleep problems in Step 2 resulted in a statistically non-significant increase in explained variability of about 4% (Δ*R*^2^ = .036, *F*[4, 123] = 1.48, *P* = .21). Adding the interaction terms in Step 3 also resulted in a non-significant increase in explained variability (Δ*R*^2^ = .042, *F*[4, 119] = 1.73, *P* = .15). Further, none of the interaction terms reached statistical significance (all *P*s > .05). The results are summarized in Table 4.

Age, sex, group, living situation and parental education level combined explained about 54% of the total variability in MVPA (*R*^2^ = .542, *F*[8, 115] = 17.04, *P* < .001). Both mid-sleep time (*β* = −.23, *P* = .015) and sleep duration (*β* = −.19, *P* = .041) were statistically significant predictors when entered at Step 2. Combined, the four focal predictors explained an additional 4% of the variability in MVPA (Δ*R*^2^ = .037, *F*[4, 111] = 2.39, *P* = .05). Adding the interaction terms in Step 3 revealed a statistically significant interaction between group and social jetlag (*β* = −.28, *P* = .012) (Table 4). This interaction shows that the effect of social jetlag is opposite for the two groups. For the normal weight group, the effect of social jetlag on MVPA is positive, while for the obese group the effect seems to be negative. Follow-up analyses of these two simple slopes showed that effect was statistically significant for the normal weight group (*b* = .28, *P* = .039) but not for the obese group (*b* = −.15, *P* = .151).

Logistic regression was used to predict participants’ odds of scoring above the clinical cut-off on emotional eating. The complete model containing all predictors was not statistically significant, *χ*^2^ (5, *N* = 170) = 8.242, *P* = .31, indicating that the model as a whole was not able to distinguish well between respondents scoring below and above the clinical cut-off. None of the individual independent variables contributed significantly to the predictive ability of the model.

## 4 | DISCUSSION

The current study found that children with obesity had significantly later sleep timing, both overall and on school nights and weekend nights separately, compared to peers with normal weight. However, sleep duration and social jetlag were not significantly different between the two groups. To our knowledge, the present study is the first to compare sleep timing in a group of obesity treatment-seeking children and adolescents with normal weight peers using objective sleep measures.

Although the amount of sleep occurring throughout the night was similar among children with obesity and children with normal weight, we found differences in the timing of when sleep occurs. There is limited research examining the association between sleep timing and BMI in school-aged children and adolescents,^9–11,13–15,17,25^ but our findings are still in accordance with the results from the majority of previous studies.^9–11,13,17^ A large cross-sectional study from Australia with 2200 participants aged 9 to 16 years^11^ found that the odds of having obesity were 1.5 times higher for adolescents with late bed/rise time than for adolescents with early bed/rise time, independent of sleep duration.

Similarly, another cross-sectional study^10^ in children and adolescents aged 8 to 17 years found that later sleep and wake times were associated with greater adiposity, regardless of sleep duration. The fact that the present and previous studies^9–11^ report an association between sleep timing and increased BMI independent of sleep duration is interesting. Sleep onset time in the present study was in total 36 minutes later for the group of children with obesity. A logical assumption, based on the growing body of evidence demonstrating an association between short sleep duration and increased BMI in children^3,4^ and the fact that the school day starts early (between 08.00 and 08.30 am), is that the group of children with obesity, due to having later sleep onset time, also would have shorter sleep duration. However, in this study we observed a compensatory delay in wake time on school days for the group of children with obesity, resulting in a sleep duration approximately the same in the two groups. The late wake-up time in the group of children with obesity makes it difficult for many in this group to reach school timely in the morning. This finding is supported by a recent meta-analysis^41^ that found that the odds of being absent from school was 54% higher among children with obesity compared to normal weight peers. In the present study it is observed that children with obesity more rarely lived with both parents. It is known that treatment-seeking children with obesity have a high degree of psychological comorbidity and often unstable family situations with increased psychological and psychosocial stress,^42^ which may influence their wake-up time. Further, it is probable that a late wake-up time might result in omitting breakfast, which is associated with weight gain in children.^43^

The later sleep and wake up time in the group of children with obesity compared to normal weight peers were consistent throughout the week. Further, both groups have approximately one-hour later mid-sleep time on weekends compared to weekdays, leading to no difference in social jetlag between the groups. This finding is inconsistent with previous research on social jetlag and BMI.^6,12,19,20^ One study reported that social jetlag was associated with higher BMI z-scores and waist-to-height ratios in adolescents aged 14 to 17 years.^20^ Similarly, another study in children aged 8 to 10 years^12^ found that social jetlag was independently associated with body fat, fat mass, fat mass index, waist-to-height ratios and BMI. Further, a study from treatment-seeking adolescents with overweight and obesity found that greater bedtime shift from weekdays to weekends were significantly associated with severity of overweight.^6^ Interestingly, a large epidemiological study with approximately 65 000 participants aged >10 years found that social jetlag did not explain significant proportions of the variance in weight in the normal BMI group, but that it was positively associated with weight increase in the overweight group.^19^ The lack of group difference in social jetlag in the present study could be explained by previous research showing that social jetlag is prevalent in adolescents across the whole weight spectrum since as many as 88% of adolescents report going to bed later on weekend nights than school nights, and 44% of high school students report a two or more hour difference in bedtimes on free nights and school nights.^44^

Additionally, it is a concern that on school nights 22.6% of the children fall below the scientific consensus-based cut off <7 hours of sleep,^35^ in terms of the many well documented adverse physical and mental health outcomes associated with insufficient sleep in children and adolescents aged 5 to 17 years.^45^ The trend of sleeping less than recommended during childhood years is apparent in several countries.^46,47^ However, only one recent study has provided data on the prevalence and stability of objectively measured insufficient sleep (<7 hours) throughout childhood.^48^ That study^48^ found that at age 12 years 14% of the children slept less than 7 hours on average, in accordance, the present study found that 13.1% of the children slept less than 7 hours on average.

Further, an aim of the present study was to examine whether sleep duration and sleep timing could explain variation in obesogenic behaviours among children and adolescents across the weight spectrum. One interesting study in this context is a recent cross-sectional investigation of the association between sleep timing with diet and physical activity in children between 9 to 11 years, using objectively measured sleep and physical activity where no significant difference in BMI or BMI *z*-score between children with late and early sleep timing was found.^14^ However, the researchers found that children with early sleep timing had healthier eating patterns, and spent more time in MVPA than children with late sleep timing, suggesting that sleep timing has less to do with BMI and more to do with behaviours that subsequently and over time may impact BMI.^14^ It is also possible that having a late sleep timing results in being awake when the internal circadian timing system favours sleep. A discrepancy between actual sleep timing and circadian rhythms may result in alterations in metabolic processes, that in the long run may have a negative effect on weight status.^23,24^

In the current study, screen time was associated with higher BMI SDS and age, as well as later sleep timing. Sleep duration did not explain a significant proportion of the variance in screen time. Additionally, MVPA was found to be inversely associated with BMI *z*-scores, age, sleep duration and sleep timing. Previous research relating sleep duration and timing with time in MVPA reports mixed results. Cross-sectional studies have contradicted each other with positive,^11,14^ negative^25^ and no significant findings.^2^ The majority of studies on this topic find that both shorter sleep duration and later sleep timing are associated with more screen time.^2,49^ However, the present results mirror those of a previous study by Olds et al^11^ who found that children with later bedtime/later wake-time engaged in less MVPA and in more screen time compared to a group of early bedtime/early wake-time children, despite having similar sleep duration.^11^ It is of interest that for the normal weight group in the current study, we found that more social jetlag was associated with increased MVPA, however, for the obese group this relationship was negative.

Finally, no associations between emotional eating and the sleep measures were found in the present study. Previous research links both sleep duration and sleep timing to poorer diets (higher energy intake and poor eating habits) in children.^9,13–16,50,51^ The few studies that have investigated sleep timing in relation to diet in children have consistently reported that later sleep timing is associated with poorer diets independently of sleep duration.^9,13–16^ Further, it is interesting that later bedtime seems to be associated with delayed time of the first meal of the day (which implies skipping breakfast) independent of sleep duration.^52^ To our knowledge, the present study is the first that specifically has investigated emotional eating in relationship to sleep timing.

The present study has several strengths and limitations that should be noted. Assessing sleep with objective measure using seven consecutive 24-hours recordings was a major asset, given that previous studies mainly have resorted to self-reported or parent-reported bed/wake-time, or sleep timing preferences (sleep chronotype) as opposed to observed sleep timing behaviours. A limitation of the present study is the lack of sleep diaries as a support to the scoring of the actigraphy recordings. However, 30% of the actigraphy recordings were scored by two independent observers to ensure inter-rater reliability, and a standardised scoring protocol was used. The low percentage of missing data, for self- and objectively measured, strengthen the findings from the present study. However, the cross-sectional nature puts restrictions on inferences about directionality and causality, and there is always a possibility for residual confounding in observational studies. Further, wrist-worn accelerometers have a lower accuracy in estimating physical activity when compared to hip worn accelerometers, which is a possible limitation of the present study. Nevertheless, both locations have been found acceptable for use in children and adolescents, but placement on the wrist has shown to have better compliance.^53^ The 10 hours of wake time between 8 am to 9 pm required for inclusion in the analyses of time in MVPA could be a possible limitation as some children may engage in physical activity outside this time frame, but is still in accordance with previously used scoring protocols for objectively measured physical activity.^36^ Inclusion of the 16 participants with late wake-up time who were excluded due to this requirement may have provided an even stronger association between time in MVPA and sleep timing. In addition, it is possible that emotional eating is an insufficient measure related to sleep timing as diet quality, calorie intake and eating patterns may be more relevant in this context. Finally, the parent-reported data on sleep problems and screen time are a limitation of the present study because of potential parent - child discrepancy in perception of sleep problems and actual screen time. Further, the question about sleep problems does not differentiate between types of sleep disturbances and there is no separation of school (mostly used for educational purposes) and leisure time (mostly used for entertainment purposes) screen use.

In conclusion, later sleep timing was related to obesogenic behaviours in children and adolescents and may represent a risk factor for obesity independent of sleep duration. These findings highlight the importance of including other aspect of sleep, in addition to duration, when conducting research and clinical work related to childhood obesity. Future longitudinal and intervention studies, with objective measurers of sleep, are warranted to better understand the association between sleep timing and childhood obesity and more studies should be devoted to understanding the underlying mechanisms of the sleep-obesity link.

## Figures and Tables

**TABLE 1:** Characteristics of the study population according to weight group

	Obesity	Normal weight	*P* value**
Total (N)	85	85	
Age (mean, SD)	12.1 (2.9)	12.0 (2.8)	.86
Range	5.9–17.1	5.8–16.4	
Sex: girls (%)	50 (58.8%)	50 (58.8%)	
BMI z-score mean (SD)	2.91 (0.45)	−0.24 (0.24)	**<.001**
Parent reported data			
Number with survey data^a^	85	65	
Mother born in Norway (%)	89.4%	97.0%	.07
Father born in Norway (%)	84.5%	93.9%	.07
Parents living together (%)	56.5%	89.4%	**<.001**
Living with siblings (%)	69.4%	95.5%	**<.001**
Father, full time work (%)	67.8%	90.9%	**.02**
Father, part time work (%)	1.2%	0.0%	**.02**
Mother, full time work (%)	47.6%	78.7%	**.02**
Mother, part time work (%)	19.0%	9.1%	**.02**
Father, completed education (%)			
Elementary school	20.5%	0.0%	
High school	41.0%	45.4%	
College/University ≤4 years	19.2%	29.7%	
College University >4 years	11.5%	23.4%	**.001**
Mother, completed education (%)			
Elementary school	11.9%	3.0%	
High school	46.4%	28.8%	
College/University ≤4 years	20.2%	34.8%	
College University >4 years	20.2%	33.3%	**.001**

Abbreviations: BMI, body mass index; SD, standard deviation.

a The percentages reported below this line are based on the number of returned questionnaires.

** *P* values from a chi-square test for categorical data, and a *t* test for continuous data; Statistically significant p values (*P* < .05) are marked in bold.

**TABLE 2:** Comparison of children with severe obesity and normal weight on sleep outcomes

Sleep outcome	Children with obesity Mean (SD)	Children with normal weight Mean (SD)	Group difference Mean	*P* value	Effect size (d)
7 day mean					
Mid-sleep time	3:37:50 (1:14:07)	3:02:08 (0:47:41)	0:35:42	**<.001**	0.57
Sleep onset time	23:23:17 (1:33:10)	22:44:22 (1:04:29)	0:38:55	**.002**	0.49
Wake-up time	7:53:39 (1:03:41)	7:20:28 (0:38:39)	0:33:10	**<.001**	0.63
School days/nights					
Mid-sleep time	3:22:21 (1:10:22)	2:46:46 (0:45:30)	0:35:35	**<.001**	0.60
Sleep onset time	23:03:27 (1:29:20)	22:25:04 (1:04:12)	0:38:22	**.002**	0.49
Wake-up time	7:40:50 (0:58:00)	7:10:00 (0:39:19)	0:30:50	**<.001**	0.62
Weekends					
Mid-sleep time	4:18:07 (1:33:40)	3:39:12 (0:59:20)	0:38:54	**.002**	0.50
Sleep onset time	00:12:14 (1:55:58)	23:31:37 (1:18:04)	0:40:36	.009	0.41
Wake up time	8:27:36 (1:31:21)	7:47:03 (0:53:43)	0:40:33	**.001**	0.54
Sleep duration, 7 day mean	7:48:31 (0:46:42)	7:52:02 (0:41:50)	−0:03:30	.608	−0.08
Sleep duration, school nights	7:39:19 (0:58:10)	7:43:01 (0:51:52)	−0:03:41	.664	−0.07
Sleep duration, weekend nights	8:09:25 (1:00:25)	8:13:36 (0:50:40)	−0:04:11	.628	−0.07
Social jetlag	00:54:32 (00:52:48)	00:52:12 (00:37:12)	0:02:24	.720	0.04

*Note:* All sleep outcomes are reported as hours: minutes: seconds. The *t* tests were evaluated using Bonferroni adjusted alpha levels of 0.004 per test (.05/13). Statistically significant *P* values in bold.

Abbreviations: d, Cohen’s d; SD, standard deviation.

**TABLE 3:** Correlations between sleep behaviour and screen time and MVPA

	Screen time	MVPA	Mid-sleep time	Sleep duration	Social jetlag
Mid-sleep time	0.458***	−0.536***			
Sleep duration	−0.267**	0.293***	−0.529***		
Social jetlag	0.170*	−0.113	0.292***	−0.114	
Sleep problems^a^	0.121	−0.051	0.246**	−0.181*	−0.009

Abbreviation: MVPA, moderate-to-vigorous physical activity.

a Correlations involving the dichotomous sleep-problems variable are point-biserial correlations, otherwise the table shows the Pearson product-moment correlations.

* *P* < .05.

** *P* < .01.

*** *P* < .001.

**TABLE 4:** Hierarchical multiple regression predicting screen time and MVPA

	Screen time (n = 136)			MVPA (n = 124)		
	B	*R* ^2^	Δ*R*^2^	B	*R* ^2^	Δ*R*^2^
Step 1		.212***			.542***	
Group	.16			−.39***		
Age	.42***			−.58***		
Sex	−.03			−.19**		
Mothers’ education						
Intermediate	.06			−.00		
Higher	.17			.03		
Fathers’ education						
Intermediate	.01			−.02		
Higher	−.21*			.13		
Parents live together	.02			.09		
Step 2		.248***	.036		.579***	.037
Group	.10			−.34***		
Age	.29*			−.57***		
Sex	−.02			−.19**		
Mothers’ education						
Intermediate	.06			.01		
Higher	.15			.05		
Fathers’ education						
Intermediate	.02			−.04		
Higher	−.18			.11		
Parents live together	.02			.09		
Mid-sleep time^a^	.26*			−.23*		
Sleep duration^a^	.08			−.19*		
Social jetlag	.05			.03		
Sleep problems	−.03			.04		
Step 3		.290***	.042		.617***	.038*
Group	.03			−.38***		
Age	.18			−.57***		
Sex	−.02			−.18**		
Mothers’ education						
Intermediate	.06			.02		
Higher	.17			.06		
Fathers’ education						
Intermediate	.01			−.03		
Higher	−.19			.11		
Parents live together	.03			.07		
Mid-sleep time	.32			−.32*		
Sleep duration	−.12			−.30*		
Social jetlag	−.16			.23*		
Sleep problems	−.22			−.04		
Group × mid-sleep time	.03			.17		
Group × sleep duration	.18			20		
Group × social jetlag	.26			−.28*		
Group × sleep problems	.26			.11		

*Note:* Group is coded normal weight = 0 and obese = 1. Sex is coded 0 = male and 1 = female. Parents living together is coded 0 = no and 1 = yes. Lower education is the base category.

Abbreviation: MVPA, moderate-to-vigorous physical activity.

a Seven day averages are used for sleep duration and mid-sleep time.

* *P* < .05.

** *P* < .01.

*** *P* < .001.

## Abbreviations:

BMI: body mass index
DEBQ: child, the Dutch eating behavior questionnaire child version
IOTF: International Obesity Task Force
MVPA: moderate-to-vigorous physical activity
NSF: National Sleep Foundation
SD: standard deviation
SDS: standard deviation score
